# Systemic Coagulation Markers Especially Fibrinogen Are Closely Associated with the Aggressiveness of Prostate Cancer in Patients Who Underwent Transrectal Ultrasound-Guided Prostate Biopsy

**DOI:** 10.1155/2021/8899994

**Published:** 2021-01-13

**Authors:** Fang-Ming Wang, Nian-Zeng Xing

**Affiliations:** Department of Urology, National Cancer Center/National Clinical Research Center for Cancer/Cancer Hospital, Chinese Academy of Medical Sciences and Peking Union Medical College, No. 17, Panjiayuan South Li, Chaoyang District, Beijing 100021, China

## Abstract

**Objective:**

It has been well elucidated that multiple types of cancers are at high risk of thrombosis. Several studies have indicated the prognostic value of fibrinogen (Fib) and D-dimer (DD) in prostate cancer (PCa). However, it remains unclear regarding the association of the comprehensive coagulation markers with the clinicopathological features of PCa.

**Methods:**

A total of 423 pathologically diagnosed patients with PCa were consecutively collected and stratified as low-intermediate-risk or high-risk groups. The association of coagulation parameters including Fib, DD, prothrombin (PT), activated partial thromboplastin time (APTT), thrombin time (TT), and antithrombin III (AT-III) with clinicopathological features was determined by univariate and multivariate logistic regression analyses.

**Results:**

The levels of Fib, DD, and PT were significantly higher in the high-risk group (*p* < 0.001, *p* < 0.001, and *p* = 0.043, resp.), while APTT, TT, and AT-III were similar between two groups (*p* > 0.05, all). Univariate logistic regression analysis demonstrated that Fib, DD, and PT were all positively correlated with high-risk PCa (OR = 2.041, *p* < 0.001; OR = 1.003, *p* < 0.001; OR = 1.247, *p* = 0.044). Nonetheless, after adjusting for PSA, grade, and stage, Fib (T3 vs. T1, OR = 15.202, 95% CI: 1.725-133.959, *p* = 0.014) but not DD or PT was the unique independent factor associated with high-risk PCa in the multivariate regression analysis.

**Conclusions:**

Our study firstly revealed that Fib but other coagulation markers was independently associated with the severity of PCa, suggesting Fib might be useful in PCa risk stratification beyond PSA, stage, and grade.

## 1. Introduction

The morbidity and mortality of prostate cancer (PCa) are increasing in recent years all over the world. In China, the proportion of patients with advanced and metastatic PCa when primarily diagnosed is higher than that in western countries [1]. Therefore, it is crucial to explore potential risk factors to more accurately evaluate the severity of PCa, which may help to early identify high-risk patients in a Chinese population.

Currently, the combination of preoperative prostate-specific antigen (PSA) level, stage, and grade is commonly applied to evaluate the severity of PCa in clinical practice [2]. However, the three parameters could only indicate the inherent malignant degree of PCa cells and local aggressiveness. Hence, searching indicators that could directly reflect the systemic turbulence induced by PCa status would be beneficial in risk stratification in the real-world clinical settings.

Plenty of evidence has shown that cancer was closely associated with hemostasis [3]. Malignancy could activate the coagulation system and promote thrombosis [4]. So far, among multiple coagulation parameters, most relevant studies focused on fibrinogen (Fib) or D-dimer (DD) in different kinds of cancers including PCa. It has been reported that PCa patients with high plasma Fib levels were prone to have higher PSA levels, grade, incidence of metastasis [5, 6], and poor prognosis after radiotherapy or androgen deprivation therapy (ADT) [7, 8]. Besides, limited studies have reported that DD was associated with higher grade and increased risk 1of mortality in patients with PCa [5, 9]. However, these studies mainly focused on certain coagulation markers such as Fib or DD and did not involve other relating parameters. Till now, only one study conducted by Alevizopoulos et al. has comprehensively investigated the association between coagulation markers and clinicopathological features of PCa [5]. Nonetheless, the sample size was relatively small (56 PCa cases), and they only separately analyzed the relationship between coagulation factors with stage, grade, and PSA, which could not systematically and comprehensively evaluate the severity risk of PCa.

Hence, the aim of the current study was to identify the association of comprehensive coagulation parameters with the severity of PCa assessed by the risk stratification system in the Chinese patients with PCa.

## 2. Methods

### 2.1. Study Population

Patients who had undergone transrectal ultrasound-guided prostate biopsy and been pathologically diagnosed as PCa at our hospital were collected between January 2012 and October 2019.

The exclusion criteria of the study were the presence of a medical history of other malignancies or coagulation-related diseases, severe hepatic and/or renal insufficiency, collagen disease, significant cardiovascular disease, or receiving any anticoagulant therapy.

All clinicopathological data including coagulation parameters were collected from medical records. All prostate biopsy specimens were routinely sent to the pathological department for diagnosis. Cancer grade assessment was performed according to the 2014 ISUP classification system [10]. The clinical tumor stage was evaluated based on MRI according to the American Joint Committee on Cancer (AJCC) TNM classification of malignant tumors in 2002.

### 2.2. Blood Sampling and Coagulation Parameter Measurement

Venous blood samples were taken at admission after 8-hour fasting and before any treatment including surgery, ADT, or other therapies. The concentrations of Fib, prothrombin (PT), activated partial thromboplastin time (APTT), thrombin time (TT), and antithrombin III (AT-III) were measured using a Stago auto analyzer, and the plasma DD level was determined on a Hitachi 7600D clinical chemistry analyzer according to the manufacturer's instructions.

### 2.3. Statistical Analyses

The quantitative variables were expressed as mean ± standard deviation (SD) except for DD, which was expressed as median with interquartile range, and were analyzed by Student's *t*-tests, one-way ANOVA, Mann-Whitney *U* tests, or Kruskal-Wallis tests as appropriate. The qualitative variables were expressed as numbers and percentages and were analyzed by the chi-square statistic tests. All PCa subjects were stratified into two groups: low-intermediate-risk and high-risk groups as previously reported [11]. Fib was divided into tertiles as follows: the first tertile group (T1, <2.74 g/L (33.3th percentile); *n* = 141), the second tertile group (T2, 2.75-3.41 g/L (33.3-66.7th percentile); *n* = 142), and the third tertile group (T3, >3.41 g/L (66.7-100th percentile); *n* = 140), and analyzed clinicopathological and coagulation features in different levels of Fib. Besides, DD and PT were divided into three tertiles and analyzed high-risk patient percentage and PSA levels in different levels of DD and PT, respectively. Correlations between coagulation parameters with high-risk PCa were revealed by univariate logistic regression analysis. The independent factors of high-risk PCa were further determined by multivariate regression analysis with adjustment for confounding factors including PSA, grade, and stage. The differences were considered statistically significant if a *p* value was less than 0.05. Statistical analysis was carried out using SPSS (version 19.0, Chicago, Illinois, USA).

## 3. Results

### 3.1. Baseline Characteristics

A total of 423 eligible PCa patients were recruited in the present study, with a mean age of 70.8 years. Patients were divided into two groups according to risk severity: low-intermediate-risk group (*n* = 63, 14.89%) and high-risk group (*n* = 360, 85.11%). The clinicopathological characteristics and coagulation parameters of the enrolled subjects are shown in Table 1. No significant differences in age, body mass index (BMI), hypertension, diabetes, or coronary artery disease were observed between the two groups.

Fib, DD, and PT levels of the high-risk group were significantly higher than those of the low-intermediate-risk group (*p* < 0.001, *p* < 0.001, and *p* = 0.043, respectively). However, there was no significant difference regarding APTT, TT, or AT-III between the two groups (*p* > 0.05).

### 3.2. Relation of Fib and Other Coagulation Factors with Clinicopathological Characteristics and Risk of PCa

PCa patients were stratified into three groups according to Fib level tertiles as mentioned above. Statistically significant differences in PSA, stage, grade, lymph node involvement, and metastasis were found among different groups (*p* < 0.001, *p* = 0.001, *p* < 0.001, *p* < 0.001, and *p* = 0.045, respectively). Specifically, there is a statistically significant trend that the late-stage (stage T3+T4) and high-grade (4+5) percentage increased with the elevation of the Fib level as mentioned above. Regarding the coagulation parameters, there were significant differences in DD, PT, TT, and AT-III but not APTT among the three Fib groups (Table 2).

**Figure 1:**
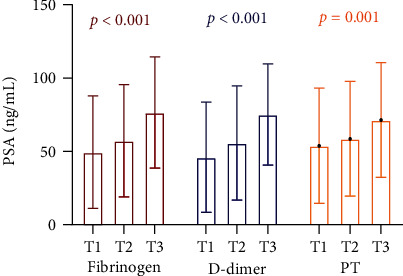
PSA levels in different tertiles of fibrinogen, D-dimer, and PT in PCa patients. PSA = prostate-specific antigen; PT = prothrombin; PCa = prostate cancer.

**Figure 2:**
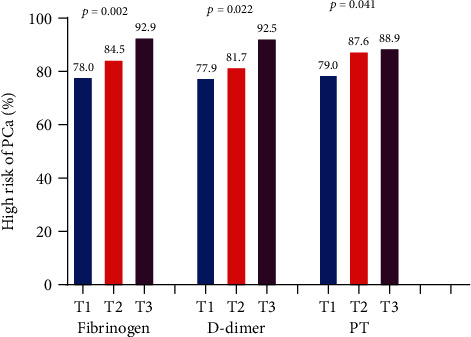
The percentages of high-risk subjects in different tertiles of fibrinogen, D-dimer, and PT in PCa patients. PT = prothrombin; PCa = prostate cancer.

Then, PCa patients were divided into three groups in terms of DD and PT level tertiles and found that PSA levels and the percentage of high-risk PCa increased with elevation of Fib, DD, and PT (Figures 1 and 2).

### 3.3. Correlations of Fib and Other Coagulation Parameters with High-Risk PCa

Univariate analysis demonstrated that Fib, DD, and PT were statistically significant parameters that were positively related to the severity of PCa. As identified in the multivariate analyses, only high Fib level remained independently and positively related to the presence of high-risk PCa after adjusting for confounding factors including PSA, grade, and stage (T3 vs. T1, OR = 3.664, 95% CI: 1.719-7.808, *p* = 0.001). However, no significant correlation between other coagulation parameters and high-risk PCa including APTT, TT, and AT-III was observed (Table 3).

## 4. Discussion

Our study for the first time revealed the association between systemic coagulation markers and severity of PCa evaluated by risk stratification system and demonstrated that coagulation parameters including Fib, DD, and PT were closely associated with the aggressiveness of PCa. Moreover, among the three markers, Fib was independently associated with the high-risk PCa. Therefore, the present study implicated that Fib might be useful in PCa risk stratification beyond PSA, stage, and grade, and it might be beneficial to be integrated into the PCa risk stratification system.

As an acute-phase protein, Fib increases during systemic inflammation, trauma, surgery, blood vessel thromboembolism, and cancer progression [12]. Previous studies claimed that high plasma Fib was associated with tumor aggression and poor prognosis in multiple kinds of cancers [13–18]. In regard to PCa, two previous studies have reported that Fib levels were positively correlated with PSA levels, T staging, and Gleason score [5, 6], which was consistent with our results. Xie et al. recently reported that pretreatment plasma Fib was positively associated with bone metastatic burden in PCa patients [19]. Besides, the elevation of Fib had been considered as a poor prognostic indicator in PCa patients who accepted radiotherapy [7] or ADT [6, 8]. Our results demonstrated that high Fib patients were more likely to have high PSA, grade, stage, lymph node involvement, and metastasis, and the general trend was consistent with the above outcome.

It is known that DD is one of the fibrin degradation products. However, elevated DD levels have been reported in patients with various cancers without clinical thrombosis [20]. Hong et al. found that DD levels of PCa patients were significantly higher than those of non-PCa patients among patients who underwent prostate biopsy [21]. Moreover, in another study, the researchers showed a significant increase of DD level in patients with advanced PCa compared with patients with localized PCa [4]. Our study divided PCa patients into three groups according to DD levels and found that PSA levels and the percentage of high-risk PCa gradually increased with the elevation of DD (from T1 to T3), which was consistent with the above results. However, as presented, DD was no longer correlated with the presence of high-risk PCa after adjusting for confounding factors in multivariate logistic regression analysis.

Our study firstly showed that PSA levels and the percentage of high-risk PCa gradually increased with the elevation of PT (from T1 to T3). Tissue factor (TF) is the initiation factor of the extrinsic coagulation pathway and plays an important role in prolonging PT. TF was expressed in multiple kinds of cancers including PCa and endothelial cells [22]. As PCa progressed, TF was upregulated and released into peripheral blood, which activated the extrinsic coagulation pathway, consumed coagulation factors, and prolonged PT. There was no relevant study regarding the direct relationship between PT and PCa at present. Only one basic research [23] revealed that androgen receptor-negative PCa cells expressed more prothrombin, possessed more thrombogenicity, and were more invasive than androgen receptor-positive cells, which supported our result.

The mechanisms of the correlation of high Fib and adverse clinicopathological features of PCa remain uncertain. There are two sources of high Fib in PCa. PCa cells can secrete tumor-associated cytokines, which stimulate the liver to produce more Fib; besides, PCa cells themselves endogenously synthesize Fib [7]. It was speculated that the ability to produce cytokines and Fib of high-risk PCa cells is much stronger than low-intermediate-risk counterparts. Conversely, Fib can promote the aggressiveness of cancer cells through several mechanisms [24]. Firstly, Fib bridges cancer cells and extracellular matrix and provides a framework to growth factors. Secondly, tumor cells have Fib receptors, which connect Fib and thus enhance the adhesion of tumor cell emboli to the endothelium of vasculature, leading to tumor spread and location out of vessels. Thirdly, Fib promotes adhesion of platelets to tumor cells, which could prevent tumor cells from the attack of CD8+T lymphocyte and natural killer cells and induce immune escape of tumor cells.

Our study demonstrated Fib was the only coagulation factor that was independently and positively correlated with the risk severity of PCa. The present study might have two potential clinical implications. Firstly, the plasma Fib could be integrated into existing PCa risk stratification systems to further improve treatment decision-making, patient consultation, and prognosis estimation. Secondly, as mentioned above, Fib plays crucial roles in cancer progression and invasion; maybe anticoagulant treatment could be applied to PCa patients with high Fib to decrease the likelihood of metastasis. Besides, anticoagulant treatment could prevent the occurrence of thrombosis and stroke in high Fib patients, which needs further study.

In conclusion, this study firstly revealed the association between coagulation profiles and clinicopathological features in PCa patients and finally found that high Fib was positively and independently associated with severity of PCa.

There were two main limitations in our study. Firstly, the prognostic significance of Fib was not evaluated in the current study. Secondly, the exact mechanism was not explored in the current analysis.

## 5. Conclusions

In conclusion, this study provided evidence of an association between comprehensive coagulation markers and clinicopathological features of PCa: patients with high Fib, DD, or PT had more adverse clinicopathological features of PCa. However, only Fib but other coagulation markers was independently associated with the severity of PCa after adjustment for other confounding factors, suggesting Fib might be useful in PCa risk stratification beyond PSA, stage, and grade.

## Figures and Tables

**Table 1:** Baseline characteristics of the study subjects according to the risk of PCa.

	All subjects (*n* = 423)	Low-intermediate risk (*n* = 63)	High risk (*n* = 360)	*p* value
*Demographic characteristics*				
Age (years)	70.8 ± 7.9	69.7 ± 6.4	71.1 ± 8.1	0.129
BMI (kg/m^2^)	24.7 ± 3.4	24.5 ± 3.4	24.3 ± 3.4	0.816
Hypertension (*n* (%))	157 (37.1)	24 (38.1)	133 (36.9)	0.888
Diabetes mellitus (*n* (%))	57 (13.5)	9 (14.3)	48 (13.3)	0.842
Coronary artery disease (*n* (%))	61 (14.4)	6 (9.5)	55 (15.3)	0.330
*Clinicopathological characteristics*				
PSA (ng/mL)	61.1 ± 39.8	10.1 ± 4.6	70.0 ± 36.4	**<0.001**
Stage				**<0.001**
T1	18 (4.3)	13 (20.6)	5 (1.4)	
T2	225 (53.2)	50 (79.4)	175 (48.6)	
T3	114 (27.0)	0	114 (31.7)	
T4	66 (15.6)	0	66 (18.3)	
ISUP grade (*n* (%))				**<0.001**
1	51 (12.1)	31 (49.2)	20 (5.6)	
2	41 (9.7)	11 (17.5)	30 (8.3)	
3	56 (13.2)	21 (33.3)	35 (9.7)	
4	101 (23.9)	0	101 (28.1)	
5	174 (41.1)	0	174 (48.3)	
*Coagulation parameters*				
Fibrinogen (g/L)	3.23 ± 1.01	2.81 ± 0.61	3.31 ± 1.05	**<0.001**
D-dimer (ng/mL)	320 (168-589)	223 (145-320)	360 (180-654)	**<0.001**
PT (s)	10.71 ± 1.32	10.40 ± 1.24	10.76 ± 1.33	**0.043**
PT (%)	117.06 ± 28.81	124.98 ± 27.93	115.68 ± 28.78	**0.018**
INR	0.93 ± 0.10	0.90 ± 0.08	0.94 ± 0.10	**0.002**
APTT (s)	32.43 ± 4.64	33.11 ± 3.95	32.31 ± 4.75	0.209
APTT ratio	1.14 ± 0.14	1.14 ± 0.13	1.14 ± 0.14	0.875
TT (s)	15.63 ± 2.35	15.64 ± 2.28	15.63 ± 2.36	0.961
TT ratio	1.04 ± 0.11	1.06 ± 0.10	1.03 ± 0.11	0.092
AT-III (%)	88.87 ± 13.44	90.36 ± 10.38	88.61 ± 13.91	0.248

Data are expressed as *n* (%), mean ± SD, or median (interquartile range). The bold value indicated statistical significance. PCa = prostate cancer; BMI = body mass index; PSA = prostate-specific antigen; ISUP = International Society of Urological Pathology; PT = prothrombin; INR = international normalized ratio; APTT = activated partial thromboplastin time; TT = thrombin time; AT-III = antithrombin-III.

**Table 2:** Clinical characteristics of study subjects according to tertiles of plasma fibrinogen levels.

	Fibrinogen tertiles			*p* value
	T1 (<2.74 g/L, *n* = 141)	T2 (2.75-3.41 g/L, *n* = 142)	T3 (>3.41 g/L, *n* = 140)	
Age (years)	70.9 ± 7.9	71.0 ± 8.0	70.7 ± 7.8	0.957
PSA (ng/mL)	49.42 ± 38.33	57.27 ± 38.35	76.61 ± 38.00	**<0.001**
Stage				**0.001**
T1	9 (6.4)	4 (2.8)	5 (3.6)	
T2	84 (59.6)	84 (59.2)	57 (40.7)	
T3	36 (25.5)	35 (24.6)	43 (30.7)	
T4	12 (8.5)	19 (13.4)	35 (25.0)	
N				
N0	114 (80.9)	100 (70.4)	71 (50.7)	**<0.001**
N1	27 (19.1)	42 (29.6)	69 (49.3)	
M				**<0.001**
M0	98 (69.5)	90 (63.4)	51 (36.4)	
M1	43 (30.5)	52 (36.6)	89 (63.6)	
ISUP grade (*n* (%))				**0.045**
1	22 (15.6)	15 (10.6)	14 (10.0)	
2	17 (12.1)	13 (9.2)	11 (7.9)	
3	24 (17.0)	16 (11.3)	16 (11.4)	
4	36 (25.5)	38 (26.8)	27 (19.3)	
5	42 (29.8)	60 (42.3)	72 (51.4)	
*Coagulation parameters*				
D-dimer (ng/mL)	231 (143-400)	293 (162-470)	520 (285-1050)	**<0.001**
PT (s)	10.56 ± 1.26	10.51 ± 1.18	11.07 ± 1.44	**<0.001**
PT (%)	118.90 ± 29.44	121.26 ± 26.78	110.96 ± 29.34	**0.007**
INR	0.92 ± 0.10	0.92 ± 0.09	0.96 ± 0.11	**<0.001**
APTT (s)	32.51 ± 5.04	32.33 ± 4.55	32.44 ± 4.34	0.948
APTT ratio	1.15 ± 0.15	1.12 ± 0.13	1.14 ± 0.13	0.174
TT (s)	16.40 ± 2.17	15.27 ± 2.12	15.22 ± 2.55	**<0.001**
TT ratio	1.09 ± 0.11	1.03 ± 0.11	0.99 ± 0.09	**<0.001**
AT-III (%)	85.83 ± 13.42	87.99 ± 12.37	92.86 ± 13.64	**<0.001**

Data are expressed as *n* (%), mean ± SD, or median (interquartile range). The bold value indicated statistical significance. PSA = prostate-specific antigen; ISUP = International Society of Urological Pathology; PT = prothrombin; INR = international normalized ratio; APTT = activated partial thromboplastin time; TT = thrombin time; AT-III = antithrombin-III.

**Table 3:** Univariate and multivariate analyses to reveal the correlation between coagulation parameters and high risk of PCa.

	Univariate mode		Multivariate mode	
	OR (95% CI)	*p* value	OR (95% CI)	*p* value
Fibrinogen (g/L)	2.041 (1.376-3.027)	**<0.001**	2.169 (0.716-6.570)	0.171
T1 (<2.74 g/L)	Reference	Reference	Reference	Reference
T2 (2.75-3.41 g/L)	1.537 (0.840-2.814)	0.163	0.748 (0.158-3.550)	0.715
T3 (>3.41 g/L)	3.664 (1.719-7.808)	**0.001**	15.202 (1.725-133.959)	**0.014**
D-dimer (ng/mL)	1.003 (1.002-1.005)	**<0.001**	1.001 (0.998-1.004)	0.448
T1 (<211 ng/mL)	Reference	Reference	Reference	Reference
T2 (212-469 ng/mL)	1.268 (0.692-2.321)	0.442	0.313 (0.070-1.394)	0.127
T3 (>469 ng/mL)	3.507 (1.385-8.875)	**0.008**	3.139 (0.310-31.779)	0.333
PT (s)	1.247 (1.006-1.546)	**0.044**	0.695 (0.379-1.273)	0.239
PT (%)	0.989 (0.981-0.998)	**0.019**	1.000 (0.977-1.024)	0.981
INR	50.64 (2.67-957.84)	**0.009**	0.072 (0.000-257.72)	0.529
T1 (<9.9 s)	Reference	Reference	Reference	Reference
T2 (10.0-11.4 s)	1.873 (0.991-3.542)	0.053	2.059 (0.458-9.256)	0.346
T3 (>11.4 s)	2.124 (1.086-4.154)	**0.028**	0.476 (0.079-2.876)	0.419
APTT (s)	0.964 (0.911-1.021)	0.209		
APTT ratio	0.855 (0.121-6.032)	0.875		
TT (s)	0.997 (0.890-1.118)	0.962		
TT ratio	0.090 (0.006-1.377)	0.084		
AT-III (%)	0.990 (0.971-1.010)	0.342		

Logistic regression analyses were performed. The multivariate model was adjusted for PSA, ISUP grade, and stage. The bold value indicated statistical significance. PCa = prostate cancer; PT = prothrombin; INR = international normalized ratio; APTT = activated partial thromboplastin time; TT = thrombin time; AT-III = antithrombin-III.

## Data Availability

All data used during the study are available from the corresponding author by request.
